# Investigation of factors influencing the implementation of two shared decision-making interventions in contraceptive care: a qualitative interview study among clinical and administrative staff

**DOI:** 10.1186/s13012-019-0941-z

**Published:** 2019-11-09

**Authors:** Sarah Munro, Ruth Manski, Kyla Z. Donnelly, Daniela Agusti, Gabrielle Stevens, Michelle Banach, Maureen B. Boardman, Pearl Brady, Chrissy Colón Bradt, Tina Foster, Deborah J. Johnson, Judy Norsigian, Melissa Nothnagle, Heather L. Shepherd, Lisa Stern, Lyndal Trevena, Glyn Elwyn, Rachel Thompson

**Affiliations:** 1 Department of Obstetrics and Gynaecology, Faculty of Medicine, University of British Columbia, E204 - 4500 Oak Street, Vancouver, BC V6H 3N1 Canada; 2 Centre for Health Evaluation and Outcome Sciences, University of British Columbia, 588 - 1081 Burrard Street, St. Paul’s Hospital, Vancouver, BC V6Z 1Y6 Canada; 3 Society of Family Planning, 225 South 17th Street, Suite 2709, Philadelphia, PA 19103 USA; 4 Dartmouth College, Level 5 Williamson Translational Research Building, One Medical Center Drive, Lebanon, NH 03756 USA; 5 Dartmouth College Health Service, 7 Rope Ferry Rd, Hanover, NH 03755 USA; 6 Patient Partner, Tyrone, NY USA; 7 Patient Partner, New York, NY USA; 8 Patient Partner, Greenwich, CT USA; 9 Dartmouth-Hitchcock Medical Center, One Medical Center Drive, Lebanon, NH 03756 USA; 10 Our Bodies Ourselves, P.O. Box 590403, Newton Center, MA 02459 USA; 11 Department of Family and Community Medicine, University of California San Francisco, Natividad Medical Center, 1441 Constitution Blvd, Salinas, CA 93906 USA; 12 Faculty of Medicine and Health, The University of Sydney, Edward Ford Building (A27), Fisher Road, Camperdown, NSW 2006 Australia; 13 Planned Parenthood Northern California, 2185 Pacheco St, Concord, CA 94520 USA

**Keywords:** Contraception, Shared decision-making, Decision aids, Decision support techniques, Question prompt lists, Implementation, Organizational culture, Interviews, Program evaluation, Theoretical Domains Framework

## Abstract

**Background:**

There is limited evidence on how to implement shared decision-making (SDM) interventions in routine practice. We conducted a qualitative study, embedded within a 2 × 2 factorial cluster randomized controlled trial, to assess the acceptability and feasibility of two interventions for facilitating SDM about contraceptive methods in primary care and family planning clinics. The two SDM interventions comprised a patient-targeted intervention (video and prompt card) and a provider-targeted intervention (encounter decision aids and training).

**Methods:**

Participants were clinical and administrative staff aged 18 years or older who worked in one of the 12 clinics in the intervention arm, had email access, and consented to being audio-recorded. Semi-structured telephone interviews were conducted upon completion of the trial. Audio recordings were transcribed verbatim. Data collection and thematic analysis were informed by the 14 domains of the Theoretical Domains Framework, which are relevant to the successful implementation of provider behaviour change interventions.

**Results:**

Interviews (*n* = 29) indicated that the interventions were not systematically implemented in the majority of clinics. Participants felt the interventions were aligned with their role and they had confidence in their skills to use the decision aids. However, the novelty of the interventions, especially a need to modify workflows and change behavior to use them with patients, were implementation challenges. The interventions were not deeply embedded in clinic routines and their use was threatened by lack of understanding of their purpose and effect, and staff absence or turnover. Participants from clinics that had an enthusiastic study champion or team-based organizational culture found these social supports had a positive role in implementing the interventions.

**Conclusions:**

Variation in capabilities and motivation among clinical and administrative staff, coupled with inconsistent use of the interventions in routine workflow contributed to suboptimal implementation of the interventions. Future trials may benefit by using implementation strategies that embed SDM in the organizational culture of clinical settings.

Contributions to the literature

- Shared decision-making is widely advocated in several nations to promote patient-centered care for contraceptive choices. There is limited evidence, however, on how shared decision-making interventions are used by health care professionals in contraceptive care.
- Through semi-structured interviews guided by an implementation theory, the Theoretical Domains Framework, we identified that staff members’ use of shared decision-making interventions was a complex process. Implementation required individual and organizational capability, opportunity, and motivation over a sustained period of time.
- Our theory-driven qualitative results fill a critical gap in the literature on the feasibility and usability of shared decision-making interventions.

## Background

Shared decision-making (SDM) is a process in which providers and patients exchange information, deliberate about available options together, identify the patient’s preferences, and incorporate those preferences in choosing the option or treatment plan [1]. In contraceptive care, SDM has the potential to improve the quality of patient-provider communication, promote patient autonomy, and enhance health and well-being by supporting the patient to choose the contraception option that matches their informed preferences.

SDM is also a model for operationalizing World Health Organization recommendations to offer “evidence-based, comprehensive information, education and counselling to ensure informed choice,” so that “every individual is ensured an opportunity for their own use of modern contraception…without discrimination” [2].

Patients who reported that “the provider and me together” decided what contraceptive method they would use were more satisfied with the decision-making process than those who reported other roles in decision-making [3]. Other research has found that SDM in contraceptive care is suited to the intimacy and complexity of this particular decision [4]. However, despite the suggested benefits of SDM interventions, and increasing calls from policy makers to use SDM as a strategy to promote patient-centered care [5–7], SDM has not been widely implemented in contraceptive care nor in other settings.

Implementation researchers have attempted to identify systematically the behaviors that may influence SDM adoption at the individual, organizational, and policy level [7–14]. For instance, in an evaluation of implementation of cancer screening SDM interventions in 12 California primary care practices, the clinician’s role was observed to be the most important factor for implementation, in combination with supportive infrastructure and the practice’s dedication to the goal of SDM [15]. However in the absence of supportive infrastructure (such as an electronic system for automatically mailing decision aids to eligible patients), implementation may be more challenging and require behavioral interventions that encourage adoption at the individual or team level [16–18].

The aim of this qualitative study was to explore the feasibility and acceptability of two interventions for facilitating SDM about contraceptive methods with a particular focus on factors that influenced their implementation by clinical and administrative staff. The study was embedded within a 2 × 2 factorial cluster randomized controlled trial (RCT), the Right For Me study, and conducted in 16 primary care and reproductive healthcare clinics in the Northeast United States [19].

## Methods

### Design

This qualitative study involved semi-structured, one-on-one telephone interviews with staff at clinics involved in the Right For Me trial (ClinicalTrials.gov Identifier: NCT02759939). Methods for the trial are published elsewhere [19]. This study was approved by the Dartmouth College Committee for the Protection of Human Subjects (STUDY00029945).

### Theoretical framework

This qualitative study was informed by the Theoretical Domains Framework (TDF), an integrative framework based on psychological theories and key theoretical constructs related to behavior change [20, 21]. In operationalizing the TDF for this research, the study team used the framework in the design, data collection, and analysis of the qualitative investigation of the implementation, acceptability, feasibility, and sustainability of the Right For Me interventions.

The TDF consists of 14 domains describing processes underlying successful behavior change. These domains map onto the capability, opportunity, motivation-behavior (COM-B) implementation model developed by Michie and colleagues [22]. In this “behavior system,” capability, opportunity, and motivation interact to generate behavior, which in turn feeds back and influences those system components: “the skills necessary to perform the behaviour, a strong intention to perform the behaviour, and no environmental constraints that make it impossible to perform the behavior” [22].

### Interventions

The two interventions comprised a patient-targeted intervention (video + prompt card) and a provider-targeted intervention (decision aids + training) in English and Spanish. These interventions are described in detail elsewhere [19] and summarized in Table 1.

**Table 1:** Right For Me shared decision-making (SDM) interventions

Intervention		Description	Intended use	Supplies provided
Patient-targeted	Video	• Video advocating patient question-asking (2:00–3:00 min) • English and Spanish versions; with and without captions	Viewed by patient at clinic before visit	• Tablet computers pre-programmed with video and with affixed instructions • Tablet chargers and cases • Headphones • Cleaning wipes
	Prompt card	• Small card reinforcing questions to ask • English and Spanish versions	Taken by patient at clinic before visit	• Cards • Display stands
Provider-targeted	Decision aids	• Set of seven one-page decision aids on contraceptive methods • English and Spanish versions	Used by health care providers with patients during visit	• Tear pads of decision aids • Desktop or wall-mounted display stands
	Training	• Training video on SDM and the decision aids (4:20 min) • Written guidance on SDM and the decision aids	Viewed by health care provider in advance of implementing decision aids (and as needed)	• Online (password-protected) access to training

### Implementation context and strategy

The 16 participating clinics were located in the northeastern United States, provided contraceptive counseling, and included Planned Parenthood affiliated clinics. Four were randomly allocated to the control arm (usual care), four to the patient-targeted intervention, four to the provider-targeted intervention, and four to both interventions. The trial was conducted during a 9-month period, with interventions implemented during the final 6 months.

Each clinic had an identified contact whose role as a senior staff member was to liaise with the research team and facilitate implementation of the interventions in their clinic. At the outset of the trial, a member of the research team (KD) visited each clinic and provided a group orientation on the trial objectives, scope, and data collection procedures. After clinics were randomized to trial arms, those clinics assigned to deliver one or more interventions received a similar, follow-up group orientation about the intervention(s). This orientation was facilitated by a presentation slide deck (see Additional file 1) and included instruction on intervention objectives, target audience, supplies provided, parties responsible for implementation-related activities, and intervention maintenance. Rather than providing strong direction on how to integrate interventions into the clinic workflow, this orientation provided examples of possible approaches and encouraged each clinic to collaboratively develop their own implementation strategy, considering the clinic workflow and other routinely used patient or counseling materials. The slide deck remained accessible to clinics via the trial website.

### Interview participants

Participants included clinical and administrative (e.g., front desk) staff aged 18 years or older who worked in one of the 12 intervention arm clinics, had email access, and consented to being audio-recorded. Depending on the clinic, physicians, physician assistants, nurse practitioners, and/or healthcare associates provided contraceptive counseling. A sampling frame was developed by KD identifying each study clinic, the names, demographics, and roles of staff in each clinic, and the project contact. The 12 clinics assigned to an intervention arm had approximately 70 staff potentially involved in implementation.

Each project contact contacted their colleagues directly and via posters in the clinic spaces and asked permission to share their email addresses with the research staff. Interested staff were emailed the study information and invited to participate in one telephone interview. Recruitment and interviews were conducted by a qualitative researcher (SM), who sampled participants first based on their role in the clinic (clinical or administrative) by referencing the sampling frame, and then purposively to seek variation in age, gender, race, profession, and years of experience. We also sought extreme case examples of clinic staff that reported having no memory of seeing or using the interventions despite having a clinic role where they would have been expected to have used them as part of their work. We did so by returning to the sampling frame and identifying participants who had either left their position or joined the clinic during the study period and were likely to be less familiar with the interventions.

### Study materials

The interview guide was based on the TDF domains, constructs, and definitions provided by Michie et al. [23] and Cane et al. [20]. Development consisted of three stages: (1) drafting a preliminary list of open-ended questions that explored each of the 14 TDF domains and a 7-item demographic questionnaire, (2) adding probes that explored domain constructs, and (3) pilot testing with three health professionals from the Right For Me research team for comprehensibility and relevance. At each development stage, additional feedback was sought from the research team.

### Data collection

Telephone interviews were conducted by SM from January to April 2017, immediately after the intervention implementation period. SM was not involved in the design or data collection of the trial or in the development of the interventions undergoing evaluation and had no relationships with the staff invited to participate. Participants were compensated with a $30 Amazon gift card. Data collection continued until (a) all participants in the sampling frame had an opportunity to respond to the request for an interview, (b) the sample demonstrated sufficient variation, and (c) data saturation was achieved (the interviews did not generate new insights regarding implementation factors). Interviews were audio-recorded and transcribed by a professional transcription service. Each was assigned a numeric identifier (e.g., “001”) and minor edits were made to remove potentially identifying information about staff and their clinics.

### Data analysis

Thematic analysis principles [24] were used to guide data analysis, which sought to identify the domains most relevant to the implementation, feasibility, acceptability, and sustainability of the interventions in practice. SM led the analysis with support from two public health researchers (KD, RM) and a nurse-researcher (DA). Each researcher first reviewed one quarter of the transcripts while listening to the audio recordings to become immersed in the data, check the transcription for accuracy, and remove names and other potential identifiers. Verification strategies were pursued throughout to ensure validity and avoid subjective bias, including having multiple researchers involved in codebook development and analysis, and constant comparison. We kept memos throughout to facilitate concurrent data collection and analysis, to maintain a data trail, and document our interpretive choices.

#### Codebook development

##### Codebook development at the TDF domain level

At the outset of the study, concurrent with the development of the interview guide, we developed a codebook consisting of the a priori TDF domains and theoretical constructs [20]. We first coded a random sample of transcripts with the codebook and met to discuss our interpretations. Through discussion, we distilled the list of 84 constructs to 39 that were most relevant in influencing clinic staff behavior. A construct was deemed relevant if it was (a) frequent, (b) participants demonstrated conflicting attitudes and beliefs about the construct, and/or (c) the construct was associated with strong attitudes and beliefs [21].

##### Operationalizing context-specific descriptions

To provide consistency in our deductive coding and interpretation, we operationalized the TDF constructs into context-specific descriptions [21]. These descriptions resulted from inductive analysis of the transcripts and were iteratively refined until reliability was achieved between the coders. Our final codebook consisted of both the TDF domains (themes) and context-specific descriptions (sub-themes).

#### Coding the transcripts

The interviewer (SM) coded all transcripts using the finalized codebook, facilitated by Atlas.ti qualitative analysis software (version 1.6.0 for Mac). RM, KD, and DA then received one-third of the coded transcripts, which they independently coded in duplicate to determine if the TDF domains and constructs were interpreted consistently, and to suggest additional codes. We compared coding and resolved disagreements through iterative discussion. Discrepancies in coding were generally due to different interpretations of constructs or lapses in attention. We met as a team through regular teleconferences to discuss, compare, synthesize, and map relationships between findings; compare our findings to our theoretical framework [20, 23]; and generate interpretive insights about the data. We discussed the data collection and analysis and sought feedback on results in progress with the larger research team via our recurring monthly teleconference, one-on-one phone calls, and a face-to-face workshop with patient and stakeholder partners.

## Results

### Overview

Interviews were conducted with 29 clinic staff from 11 of the 12 intervention clinics (see Table 2). All clinic staff identified as female and were predominantly White, reflecting the demographics of the region, and majority gender of professionals in contraceptive care. The majority had a clinical (*n* = 16, 55%) or both clinical and administrative role (*n* = 4, 14%) that involved providing contraceptive counseling to patients. Interviews were 20 to 40 min in length. Staff invited to participate from Clinic 4 (decision aids + training) either did not respond to invitations or declined to participate. Reasons for declining included being too busy, feeling they had nothing meaningful to contribute, or being a recent hire to the clinic with no interaction with the interventions.

**Table 2:** Characteristics of the clinic staff participant sample (*n* = 29)

Characteristic	*N*	(%)
Gender, female	29	(100.0)
Age		
20–29	6	(20.7)
30–39	9	(31.0)
40–49	5	(17.2)
> 50	9	(31.0)
Race		
White	27	(93.1)
Other race(s)	2	(6.9)
Profession		
Health care associate/assistant/worker	7	(24.1)
Nurse practitioner/physician assistant	7	(24.1)
Managerial/administrative (e.g., Clinic manager, Executive Director)	5	(17.2)
Medical assistant	3	(10.3)
Registered nurse/licensed practical nurse	3	(10.3)
Physician/resident	2	(6.9)
Role in the clinic		
Administrative	9	(31.0)
Clinical	16	(55.2)
Both	4	(13.8)
Years since completing professional training		
0–5	10	(34.5)
6–15	3	(10.3)
16–25	11	(37.9)
26 or more	5	(17.2)
Duration of time involved in the study		
Less than the entire study period	10	(34.5)
Entire study period	19	(65.5)

Each clinic chose to implement the interventions using a different approach (see Table 3). Some clinics chose to keep the decision aids in an easily accessible space, such as on a desk or in a wall display holder in exam rooms (Clinic 1, 9–12). In most clinics, the video and prompt cards were handed to patients in the waiting room (Clinics 6, 11, 12) or private exam room (Clinic 7–10) prior to the visit.

**Table 3:** Characteristics of the clinic settings (*n* = 11)

Trial arm (# staff participants)	Clinic^a^	Size	Clinic type	Clinic aware of interventions?			How were the interventions used?	Where were the interventions kept?
				DA	V	PC		
Decision aids + training (*n* = 7)	1	S	Primary care	Y	–	–	During the appointment, the clinical staff person selected a relevant decision aid, explained it, gave it to the patient, they circled questions together, and then used it to make or defer a choice. Some patients took it home.	Hung in display holders screwed to the wall of clinic rooms.
	2	S	Reproductive health care	Y	–	–	After the patient had chosen a method, the clinical staff person went to get a method-specific decision aid, explained that it gave more information on benefits and harms, the patient read it, and then the provider answered any questions.	Hung in the hallway with all other clinic patient resources.
	3	S	Reproductive health care	Y	–	–	During the appointment, the clinical staff person selected a relevant decision aid, explained it, gave it to the patient, and wrote on it and circled questions together. Then the provider pulled out their organization’s contraceptive counseling resource to guide discussion of the benefits and harms of the patient’s chosen method.	Organized in a filing cabinet with the organization’s other contraceptive counseling resources.
Video + prompt card (*n* = 12)	5	L	Primary care	–	Y	N	Staff did not hand out the video or prompt cards. Staff put up a sign in the waiting room inviting patients to watch the video.	Both were left in the general waiting room; cards were also on each appointment desk.
	6	S	Reproductive health care	–	Y	N^b^	The front desk person handed the video tablet, and later the prompt cards, to the patient at check-in.	Both were kept at the front desk.
	7	S	Reproductive health care	–	Y	Y	The front desk person handed the video tablet and prompt card to each patient in a private waiting room, and gave verbal instructions.	Both were kept in a private, clinical waiting room.
	8	L	Reproductive health care	–	Y	U	Patients had the option to watch the video while waiting during the clinical appointment.	U
Both interventions (*n* = 10)	9	S	Reproductive health care	Y	Y	Y	The front desk person handed the video tablet to the patient to watch while waiting in the exam room. During the appointment, the clinical staff person selected a relevant decision aid, explained it, gave it to the patient, they pointed at questions together, and then used it to make or defer a choice. Some patients took it home.	Video was kept at the front desk; prompt cards left in the waiting room and clinic rooms; and decision aids kept on the exam room desks, organized with “flags” in the display holder.
	10	L	Primary care	Y	Y	N^c^	The front desk person handed the video tablet to the patient to watch while waiting in the exam room. During the appointment, a clinical staff person selected a relevant decision aid, explained it, gave it to the patient, and they discussed questions together.	Video was kept at the front desk; prompt cards were left in the waiting room; decision aids were hung in display holders screwed to the wall of clinic rooms.
	11	S	Reproductive health care	Y	Y	Y	The front desk person handed the video tablet and a prompt card to the patient to watch while in the waiting room. During the appointment, the clinical staff person selected a relevant decision aid (or took the whole pad), explained it, gave it to the patient, they circled questions together, and then used it to make or defer a choice. Then the provider pulled out their organization’s contraceptive counseling resource to guide discussion of the benefits and harms of the patient’s chosen method.	Video was kept at the front desk; prompt cards were kept at the front desk, and in the waiting and clinic rooms; the decision aids were kept in display holders in each clinic room.
	12	S	Reproductive health care	Y	Y	Y	The front desk person handed the video tablet and a prompt card to the patient to watch while in the waiting room. During the appointment, some clinical staff selected a relevant decision aid, explained it, gave it to the patient, pointed to questions together, and used it to make or defer a choice. Other staff handed out the decision aids as take-home educational material, and/or pulled out their organization’s contraceptive counseling resource to guide discussion of the benefits and harms of the patient’s chosen method.	Video and prompt cards were kept at the front desk; decision aids were kept on the exam room desks, organized in the display holder.

*DA* decision aids, *V* video, *PC* prompt cards, *S* small (< 10 staff), *L* large (> 10 staff), *Y* yes, *N* no, *U* uncertain

^a^No staff members from Clinic 4 chose to participate

^b^Staff discovered the prompt card mid-study

^c^This is based on findings from the majority of clinic staff. Only one staff person reported being aware of the prompt card

When analyzed through the lens of the Theoretical Domains Framework, we observed that each implementation approach was the result of dynamic interaction between multiple domains. It was necessary first for clinic staff to have the *capability* to implement the interventions, primarily knowledge, skills, memory, and behavioral regulation to use them routinely. Their behavior was also *motivated* by their social/professional role and identity, beliefs about consequences, and goals. Implementation was also modified by factors that influenced the *opportunity* to engage in the implementation behavior: social influences (of patients) and their environmental context and resources (see Table 4).

**Table 4:** Mapping of the Right For Me findings to the Theoretical Domains Framework (TDF)

COM-B component		TDF domain	Construct	Right For Me implementation factor
Capability	Psychological	Knowledge	Knowledge	Being aware of the interventions
			Procedural knowledge	Knowing how to use the interventions correctly
		Skills	Competence	Having the proficiency to use the interventions, acquired through training or practice
		Memory, Attention, and Decision Processes	Memory	Remembering to use the interventions
		Behavioral regulation	Action Planning	The action or process of forming a plan regarding implementing the interventions
			Behavioral regulation	Changing one’s behaviour to engage in the new practice of using the interventions
Opportunity	Social	Social influences	Social influences	Feeling influenced by interpersonal processes with patients
	Physical	Environmental context and resources	Environmental stressors	Feeling influenced by clinic workflow, time, or physical space
			Resources/material resources	Supplementing the interventions with other resources
			Organizational culture/climate	Feeling influenced by the clinic’s organizational culture
Motivation	Reflective	Social/professional role and identity	Professional role	Demonstrating professional behaviors and qualities that influence use of the intervention
		Beliefs about consequences	Outcome expectancies	Believing that use of the interventions enhances SDM, or not (or has other consequences)
	Automatic	Reinforcement	Incentives	An external stimulus that enhances or serves as a motive for implementation

### Capability

A necessary antecedent to implementation was first being aware of the interventions (“Knowledge”) and how to use them (“Procedural Knowledge”) (see Table 5 for representative quotations). While most staff were aware of the decision aids and patient video, some reported that they never watched the video or viewed the prompt card, were absent during the study team orientation, or did not notice the video and prompt card until partway through the implementation period. This led some to be unclear about the purpose of these interventions. In contrast, all clinical staff knew about the purpose of the decision aids and reported tending to use them with fidelity, following the steps provided in the online training (“Explain it, Give it, Use it”). In the context of Clinic 2, however, staff reported using the decision aid after the patient had made a choice, as a way to discuss the benefits and harms of the contraception method:

So they choose the method. We review the medical history with them. Then I’ll say, ‘Oh, we’re going to bring you upstairs to see the practitioner, or we’re going to bring you upstairs to see the nurse and I’m also going to give you our handout to go home with as well, which says a little bit more about the rare side effects and complications of the methods.’ (015, Clinic 2, Clinical role, Decision aids + Training)

Clinical staff perceived that the group orientation and decision aid training reinforced their proficiency in patient communication (“Skills”). There was vast heterogeneity in how each staff member engaged with the training; some clinics had all staff, both clinical and administrative, complete the training as a team at the outset of implementation, while others asked them to complete it during their own time, and a minority was unaware that they could have accessed the training themselves.

**Table 5:** Factors related to “Capability” and representative quotations

Construct	Right For Me implementation factor	Quotations
Knowledge	Being aware of the interventions	“I think that the videos were about different birth control options. But I am not entirely sure.” (026, Clinic 8, Clinical + administrative roles, Video + Prompt Card)
Procedural knowledge	Knowing how to use the interventions correctly	“Patients did not take the learning tools [decision aids] and write notes on them like it was suggested in the [training] video. I do not think I had but maybe one or two people do that. I do not know if people just did not want to. I do not know if people were not asking in a way that they felt comfortable … It makes me think that perhaps the staff stopped even utilizing that aspect of that suggestion over time because people were not taking them up on it.” (015, Clinic 2, Clinical, Decision aids + Training)
Competence	Having the proficiency to use the interventions, acquired through training or practice	“So if someone comes in and says, ‘I know I want birth control. I’ve never tried anything before. Where do we start?’ I’d say like, ‘Okay.’ We would grab the all methods tool … Because some of our patients have never used a birth control method before. They say ‘birth control’ and they think of the pill. And I go, ‘Well there are many more options out there. There’s not just the pill.’ We get that sheet out and sort of do a general overview. If a patient then identifies what they are looking for in a method, whether it’s something that’s long-term or something that has a really high effective rate. Or maybe they do not want any hormones. Or maybe they think they might want to be pregnant within the next year and they want something that’s going to be shorter acting because they know it’s not right now. Based on wherever that conversation goes … I would then grab the next tool [method-specific decision aid].” (013, Clinic 11, Clinical role, Both interventions)
Memory	Remembering to use the interventions	“Oh and there were some other cards actually now that I remember that had the questions. There were like the five questions or the three questions—this was a long time ago, I’m really sorry if my brain is kind of fuzzy—that I probably could have utilized those more. But patients aren’t really psyched about taking anything material to be totally honest. I mean you hand them something and you see it in the trash in the hallway.” (019, Clinic 5, Clinical role, Video + Prompt card)
Action planning	The action or process of forming a plan regarding implementing the interventions	“The biggest thing was I think creating the space for the tool, so like physically and mentally. So rearranging the rooms in a way so that we have those sort of stackable file holders, and putting all of the tear-off sheets in those in an order and in a way that made sense. Physically creating the space for cards, and where we were going to set up the iPads and the chargers. And then also just mentally sort of—we have a workflow, and just reviewing that and going over, okay, so where are you going to ask this? When a patient is checking in, at what point are you going to say we are participating in this study? Are you interested in watching a short video? How do we build that into the vocab? Because a lot of what we say is somewhat scripted, obviously in the room depending on the patient need, it’s not. But the initial introductions are, and that’s often times where the information needed to come in, so is just working with the staff to figure out what feels the most natural, how do we implement this into our everyday language routine.” (016, Clinic 9, Administrative role, Both interventions)
Behavioral regulation	Changing one’s behaviour to engage in the new practice of using the interventions	“I mean I think the biggest challenge is just changing the behavior of the people who are interacting with [patients] to incorporate one more thing. There’s a lot of things that we are always asked to remember—to screen for depression and screen for this and look for that and do all these things—and so to have one more thing to sort of hand them. Everybody out there sort of wants you to hand [patients] one more thing, and that’s the most challenging part—is sort of triaging those and changing. Like I said, we all sort of get into our routines, and so to try to remember a new thing.” (Participant 19, Clinic 5, Clinical role, Video + prompt card)

Clinical staff across all 12 clinics had a strong perception that SDM was already a part of their contraceptive counseling approach (“Professional Role”), was appropriate for their clinical context (“Organizational Culture/Climate”), and was facilitated by their use of existing contraceptive counseling resources (“Resources/Material Resources”):

We didn't need a whole lot of training, because this is what I do all the time. So, I have different decision tools, I found them useful, and I didn't – I've been doing it for 20 years ... I didn't need a lot of training to know how to use these with women. Because I also have been trained in shared decision making, and motivational interviewing, and all of that. (002, Clinic 1, Clinical role, Decision aids + Training)

Descriptions of their contraceptive counseling approaches suggested that they successfully used the decision aids to engage in components of SDM. For instance, participants from reproductive health care clinics explained that they typically placed the decision aid providing an overview of all contraceptive methods on a desk between themselves and the patient and used it as a visual aid while asking open-ended questions to elicit the patient’s preferences and identify the features of a birth control method that would suit the patient’s needs. Despite this, health care providers did not always report taking the next step of asking patients to indicate their preferences in writing (“Procedural Knowledge”). For some, this was due to perceived patient disinterest or discomfort with this step (“Social Influences”) and, in these contexts, patient influences were a factor in staff use of the decision aids: “It makes me think that perhaps the staff stopped even utilizing that aspect of that suggestion over time because people weren’t taking them up on it.” (015, Clinic 2, Clinical, Decision aids + Training).

Clinical staff perceived that getting into the habit of using the decision aids (“Behavioral Regulation”) was relatively easy because of their similarity to existing contraceptive counseling resources. However, participants explained that providing a tablet computer to patients and asking them to watch a video before their clinical visit required a change to existing routines for administrative staff. Three clinics (7, 11, and 12) got into the habit of implementing the prompt cards routinely by handing them out to each patient prior to the visit, while others clinics (5, 6, and 10) chose to place a stack of cards in the waiting room or exam rooms and did not interact with the cards thereafter. Notably, staff from clinics that did not have a plan for using the prompt cards and did not make them part of staff routines were the same ones unaware that the prompt cards existed (see Table 3).

Participants that reported gaining proficiency in how to use the interventions typically perceived clear leadership from the project contact, who acted as a liaison with the research team. For instance, after experiencing significant staff turnover, the project contact in Clinic 9 began providing one-on-one, in-person training to staff in how to use the decision aids interactively, using a role-playing technique. Other project contacts had staff review all of the interventions to create common understanding (“Knowledge”), while others worked with staff to develop a plan for how to use them (“Action Planning”).

Clinics implemented the interventions in a way aligned with their work style, contextual needs, and team dynamics (see Table 3). Most clinics developed a plan at the outset of the study for how, where, and when they would integrate the interventions into clinic workflow, mental reminders, and talking points (“Action Planning”), as one participant described:

The biggest thing was I think creating the space for the [interventions], so like physically and mentally. So rearranging the rooms in a way so that we have those sort of stackable file holders, and putting all of the tear-off sheets in those in an order and in a way that made sense. (016, Clinic 9, Administrative role, Both interventions)

Handing the interventions directly to patients and explaining their purpose was more acceptable and feasible than relying on the patient to use them if interested. In contrast, in the context of Clinic 5, participants used a passive strategy for the video and prompt card because staff perceived it was the patient’s responsibility to engage with them:

We just kind of allowed them to watch the video without any intervention on our end to say, ‘Feel free to watch this or we’d like you to watch this beforehand,’ or ‘You have to watch this on your visit.’ It was kind of freeform. (003, Clinic 5, Administrative role, Video + prompt card)

### Motivation

Clinic staff were motivated to implement the interventions if they felt that they were aligned with their existing roles and responsibilities (“Professional Role”) and added value to their work (“Reinforcement”) (see Table 6 for representative quotations). The content of the decision aids reflected what clinical staff would typically discuss with patients, and provided a textual cue to reinforce their “talking points.” One key contextual factor in Clinic 10 was that the project contact changed her implementation approach and encouraged nurses to use the decision aids as part of their responsibilities after observing that they had not adopted them into their professional role.

**Table 6:** Factors related to “Motivation” and representative quotations

Construct	Right For Me implementation factor	Quotations
Social/professional role and identity	Demonstrating professional behaviors and qualities that influence use of the intervention	“We did not need a whole lot of training, because this is what I do all the time. So, I have different decision tools, I found them useful, and I did not—I’ve been doing it for 20 years ... I did not need a lot of training to know how to use these with women. Because I also have been trained in shared decision making, and motivational interviewing, and all of that.” (002, Clinic 1, Clinical role, Decision aids + Training)
Beliefs about consequences	Believing that use of the interventions enhances SDM, or not	“I’d be curious to know what the study shows as far as patients who took the survey who also said they watch the video. It’s just a tool that was much more hands off for me. So I do not know that it’s not effective. I just did not, when patients were watching it, they were watching it by themselves in the waiting room and I do not know how it spoke to them or how they responded to it. Which is something I’m curious to hear as the study results come out. Because I’m not anti-video. I just do not see it the same way as I do the other tools.” (013, Clinic 11, Clinical role, Both interventions)
Reinforcement	An external stimulus that enhances or serves as a motive for implementation	“I know that there was one specifically where a woman came in to start on birth control pills for the first time. She was like in her early, early 20’s. And we just used that tool to kind of go over, ‘Well, this is the short-acting method. This is the long-acting method.’ She was starting college or was a college student, so she felt like a long-acting contraceptive method might be better for her, and she ended up going home with an IUD as opposed to the birth control pill, so it just felt like to her it fit her needs better. And she was able to see that clearly on that sheet – that there were long-acting methods as opposed to short-acting and that being more of a fit for her.” (020, Clinic 9, Clinical role, Both interventions)

While decision aids were perceived to help clinical staff exercise their existing responsibilities for contraceptive counseling, handing out the videos and prompt cards did not help front desk staff complete their administrative tasks, and this misalignment was a barrier to implementation (“Reinforcement”).

Administrative staff did not know what took place between patients and clinical staff after the patient had watched the video, while clinical staff reflected that they did not know which patients had watched the video in the waiting room: “I’d be curious to know what the study shows as far as patients who took the survey who also said they watch the video. It’s just a tool that was much more hands off for me” (013, Clinic 11, Clinical role, Both interventions). Staff who watched the video had some criticisms of the content, namely that the featured patient was not representative of their patients and sounded “rehearsed,” negatively impacting their motivation to implement it. In contrast, clinical staff involved in contraceptive counseling illustrated the motivating value of directly observing or experiencing a positive effect as a result of using the decision aids (“Reinforcement”). For instance, some staff shared that, after using a decision aid, patients chose a method of contraception that seemed best aligned with their preferences.

### Opportunity

The physical context of implementation (“Environmental Context and Resources”) influenced staff members’ motivation and plans to use the interventions. The implementation process was perceived to be easiest in clinics with a self-described “small team” and low caseload, and/or where staff felt they had flexible procedures and infrastructure to adapt the interventions to fit existing routines and their clinic environment (see Table 7 for representative quotations). Implementation was also facilitated in contexts where reproductive health clinics perceived that their organizational routines and priorities were aligned with the goals for the study (“Organizational Culture”). One participant clarified that because their organization follows the “same guidelines and expectations” for informed contraceptive choices “it was just easy and natural. It flow[ed] very naturally for us” (011, Clinic 3, Clinical role, Decision aids + Training).

**Table 7:** Factors related to “Opportunity” and representative quotations

Construct	Right For Me implementation factor	Quotations
Social influences	Feeling influenced by interpersonal processes with patients	“I would say that the majority of [patients] that come into the waiting room are on their own phones and computers and are not really looking around. We have a TV. We have a lot of educational things about just general health and I feel like the [patients] are not tuned into that because they are just on their phone or on their computer or reading their books.” (Participant 27, Clinic 5, Clinical role, All interventions) “Patients did not take the learning tools [decision aids] and write notes on them like it was suggested in the [training] video. I do not think I had but maybe one or two people do that. I do not know if people just did not want to. I do not know if people were not asking in a way that they felt comfortable. I do not know. We’re pretty open and a safe space for people to do whatever the heck, say whatever the heck they want. But nobody was writing anything.” (Participant 15, Clinic 2, Clinical role, Decision aids + training)
Environmental stressors	Feeling influenced by clinic workflow, time, or physical space	“Sometimes the patient would take the iPad back, like, into one of the back rooms, and they would finish watching it, while the back HCA [Health Care Associate] was getting their information into the computer. And then I had to be, like, ‘Okay, so where’s the iPad?’ It just kind of added another thing to our plate that, we already do not have a lot of time … We’ve got a lot going on … so I would say that’s mostly it. It just kind of made things a little bit clunkier.” (Participant 5, Clinic 6, Administrative role, Video + Prompt card) “I think it changed our workflow for our administrative staff the most. It really did not change my workflow at all because there was going to be counseling anyway for birth control. I mean it made it a little bit quicker in terms of people already having those sheets and being able to look them over and things like that, but I think the front staff had more of the workflow changes because they had to implement the video and then the survey at the end, and getting that into patient’s hands was a little bit trickier than our portion.” (Participant 20, Clinic 9, Clinical role, All interventions)
Resources/material resources	Supplementing the interventions with other resources	“I think the study materials are beautiful. Like they were easy to read. They were, you know, they were bright and I think very attractive … and I think that with our goals around patient access and our goals around efficiency, you know, the added layer of an additional paper form I think was cumbersome. And so the ones we did put out on the desk were the study forms. So it was, you know, when someone came in and they were looking at whether they wanted to go on pills or a Nuva or something like that, they would pull out the appropriate forms and they’d leave those out on the desk for the patient, review the pertinent information of each and leave those on the desk. But when they would go through like the contraceptive counseling, you know, reviewing the benefits, the risks, the alternatives, the warning signs, they would use our materials because they were in line with our talking points.” (Participant 28, Clinic 12, Both clinical and administrative roles, All interventions)
Organizational culture/climate	Feeling influenced by the clinic’s organizational culture	*Staff person*: We did not really have any conversations after [implementation]. There was, “This what we are using,” and just we went for it and used them. *Interviewer*: Why do you think it was so easy? *Staff person*: I think because [reproductive health organization], like I said, really follows the same guidelines and expectations, that it was just easy and natural. It flows very naturally for us.” (011, Clinic 3, Clinical role, Decision aids + Training)

Clinical staff typically felt that the decision aids fit easily into the clinic or counseling room space, and helped to make appointments shorter by creating a more focused conversation. A minority of clinical staff perceived that decision aids “trigger[ed] more questions, and therefore a 15-minute visit would tend to be 20 or 25 minutes” (017, Clinic 10, Clinical role, Both interventions). However, these staff typically found the longer visits easy to adjust to (“Environmental Stressors”). Staff that received both interventions did not perceive that having multiple components added to their implementation “load,” in part because of the division of labor between clinical (decision aids + training) and administrative (video + prompt card) staff. Rather, comments about time pressure and workload were more common among staff who were already experiencing environmental stressors, such as participating in other research studies or having unexpected staff turnover.

Staff from clinics with existing approved counseling materials used these and the decision aids together, so that one supplemented the other (“Resources/Material Resources”). Few staff felt that using multiple resources was cumbersome in counseling, because the resources were so similar. As described above, a minority of staff at reproductive health care clinics perceived that they already do SDM (“Competence”) and this attitude led them to believe that the decision aids and accompanying training were redundant (“Motivation”). No staff suggested that there were any existing materials that were replaced by or supplemented the video or prompt card.

Finally, one of the core domains that influenced implementation behavior was the “Social Influence” of patients on staff members’ routine use of the interventions. While staff perceived that patients found the decision aids acceptable, they felt that patients had minimal interest in the video and prompt cards, potentially because using them was inconsistent with typical waiting room behavior. Nonetheless, staff felt that both interventions were appropriate for their patient population, in particular for those with lower education and literacy, and those making their own health care decisions for the first time.

### Sustainability

When asked if they would want to continue using the interventions now that the trial was complete, staff reported that they would like to continue using the decision aids but had mixed feelings about the video and prompt card. Without the tablets provided by the Right For Me study, most felt that their clinic would be unlikely to continue use of the video in its current format. While staff were keen to continue using the decision aids, those affiliated with a larger organization or network also felt that future implementation decisions would have to be made “high up,” for consistency of content and branding across the organization.

## Discussion

Our findings suggest that the decision aids were more acceptable, feasible, and sustainable than the video and prompt cards. Awareness of these interventions, knowing how to use them correctly and competently, integrating the interventions into regular workflow, and having a professional role and organizational culture that supported using the interventions appeared to facilitate intervention implementation. Clinic environments, workflow, and physical space supported implementation of the decision aids, but did not facilitate use of the video and prompt card. While some facilitators are context-specific, our findings suggest that introducing interventions will not be successful without the resources required to modify existing routines and to monitor and sustain behavior change.

In clinics where implementation was explained as relatively weak, it appears that the interventions were not considered an essential professional responsibility. While integrating a video via tablet into a busy waiting room may not be a feasible strategy for facilitating SDM, integrating paper-based decision aids into clinical routines may prove more successful. However, it requires negotiating and planning as to what the task is, who does it, how it gets done, and whether it adds any real value.

Elwyn and colleagues conducted a thematic analysis of qualitative interviews embedded in the intervention phase of a trial of similar clinical encounter decision aids for treatment of knee osteoarthritis [25]. Before using the decision aid, clinicians expressed concern about time pressures, patient resistance, and patient information overload [25]. After minimal training, the same clinicians perceived that the decision aid was acceptable and helpful, and it had changed their usual way of communicating. In the USA, case studies of implementation of clinical encounter decision aids in routine care suggested that physicians may not perceive the decision aids have utility, particularly for patients with low literacy [26]. Lack of suitability for patients was not a factor that emerged from analysis of our clinic staff interviews; rather, participants suggested that patients with low literacy or limited education would benefit most from interventions that facilitate SDM.

Our findings suggest that participants in clinics that implemented both interventions did not experience implementation overload in comparison with those that were exposed to one only. Similar to a study investigating implementation of a clinical encounter decision aid for circumcision, we observed that gaining the skills to use the decision aid through practice (a learning curve) was necessary [27].

The research team overestimated some clinics’ capacity to self-organize in designing and preparing for implementation even when the interventions were conceptually aligned. However, the solution for bridging this capacity gap is unclear. The MAGIC (making good decisions in collaboration) program, which sought to implement SDM into routine primary and secondary care, similarly observed that clinical teams feel they already involve patients in decisions about their care [13]. In that program, hands-on role-playing that promoted practical skills and exercises to change embedded attitudes helped to show clinicians how SDM differed from their current practice. Changing individual SDM behavior in contraceptive care may thus require more interactive training, such as role-playing, that emphasizes both skills (Capability) and the value of the skill to the individual and their organization (Motivation).

All clinics had a strong perception that SDM was already a part of their organizational culture, and was facilitated in some clinics by use of existing educational resources. The high acceptability of the decision aids may stem from our extensive provider consultation about their content [28]. Implementing the decision aids was perceived to be a simple step at implementation (e.g., swapping their existing resources for the Right For Me interventions) but sometimes difficult to remember on a day-to-day basis. Not all staff randomized to the decision aid and training intervention reported completing the online modules or using the decision aids as intended (e.g., used them during the counseling encounter; wrote on them). A meta-analysis of six randomized controlled trials conducted in US practice settings [29] similarly observed that few clinicians used clinical encounter decision aids with fidelity. The authors of the meta-analysis observed that, after implementing the interventions, clinicians used them as intended only partially and inconsistently, and that higher fidelity was associated with increased patient knowledge and patient involvement in decision-making [29]. Such findings have led Montori and colleagues to suggest that “the answer is not in” regarding the effect of decision aids on SDM [30].

Clinics exposed to the video and prompt cards had limited awareness of the cards, and perceived that the videos were difficult to integrate into routine workflow and were of limited interest to patients. The implementation process was seen to be easiest in smaller clinics, or where staff felt they had flexible procedures and infrastructure to adapt the interventions to fit routine practice. The success of implementing these new routines was also dependent on the actions of clinic patients, who may either accept or decline to use the Right For Me interventions. Survey responses from patient participants in the trial will provide further insight into the number of patients who reported using the Right For Me interventions, and what proportion would recommend them to a friend (e.g., acceptability). Participants felt that both interventions were most appropriate for their low health literacy patients. However, these attitudes may not be supported by emerging literature. Recent investigations from Australia suggest that generic question sets alone, like those used in our video and prompt card, are not sufficient to support shared decision-making among adults with low literacy [31] and additional strategies may be required to improve understanding of SDM terms and probability concepts [32].

The video and prompt card used in this study were adapted from the “Ask, Share, Know” program previously tested and implemented in an Australian primary care setting [33]. A systematic review of the use of question prompt lists in routine practice highlighted the importance of “endorsement,” that is, when the list is not given to or mentioned by the clinician, studies demonstrate inconsistent findings with respect to patients’ question asking [34]. In our study, this construct was reflected in clinic staff “motivation.” Clinical staff were largely unaware of which patients were exposed to the video and prompt card, and wished to know so they could respond to the patients’ questions and observe whether or not they were useful to contraceptive counseling. Implementation of the video and prompt card may thus require an organization-wide or team-based training that increases clinician awareness of and motivation to engage with them.

Our study findings also suggest that there may be differences in implementation practices for patient-targeted interventions implemented by administrative staff (video + prompt card) versus those that are intended for the provider to use with the patient (decision aids). The organizational or institutional context may also play an important role. Sexual and reproductive health clinics and organizations have well-established norms, such as organization-wide counseling protocols and branding. These norms may represent a double-edged sword—they provide the capability, opportunity, and motivation for staff to engage in SDM, but may be inflexible to change.

Limitations of this study include lack of accompanying observational strategies for assessing implementation success. This meant we were unable to investigate the relationships between staff perceptions and actual implementation. We also took measures to minimize social desirability bias, but some participants may have over-reported positive and under-reported negative perceptions, attitudes, or experiences. In spite of our partnerships with and recruitment support from clinic staff at each study clinic, we received limited interest in participation from some clinicians, leading to no data for Clinic 4 and only one participating clinic staff each for Clinics 8 and 11. Findings from those clinics should be interpreted in relationship to the other settings, not individually.

The strengths of this study include our systematic application of a theoretical framework for behavior change [22] to develop SDM interventions, comprehensive evaluation of their use in routine contraceptive care, and identification of factors that influence their use. Having an independent researcher conduct and analyze the interviews mitigated potential interviewer and reporting bias. Finally, by including administrative staff in the study sample, we gathered data on the implementation experience of stakeholders across clinic organizations. Interviews with administrative staff provided critical data on the feasibility of the video and prompt card interventions, which would not have been collected through interviews with clinical staff alone.

## Conclusion

Our results suggest that clinical and administrative staff perceived the clinical encounter decision aids to be more acceptable and feasible to implement than the patient video and prompt card questions. Implementation of interventions that align with existing roles, tasks, and workflow may have greater acceptability, feasibility, and sustainability than those that require new procedures and infrastructure. We demonstrated how use of the Theoretical Domains Framework can be used to understand the factors that influence implementation of SDM, and to create interventions that are theoretically and behaviorally informed. Future studies could build on our findings of the factors that influence implementation of SDM and use the Behavior Change Wheel and COM-B frameworks to characterize and design strategies for implementing our study interventions in different settings [22].

## Supplementary information

**Additional file 1.** Orientation materials for clinics implementing the Right For Me interventions.

## Data Availability

The qualitative datasets generated and/or analyzed during the current study are not publicly available due to privacy and ethics restrictions.

## Abbreviations

COM-B: Capability, opportunity, motivation-behavior
MAGIC: Making good decisions in collaboration
PCORI: Patient Centered Outcomes Research Institute
SDM: Shared decision-making
TDF: Theoretical Domains Framework

## References

[1] Charles C, Whelan T, Gafni A (1999). What do we mean by partnership in making decisions about treatment?. BMJ..

[2] World Health Organization (2014). Ensuring human rights in the provision of contraceptive information and services: guidance and recommendations [Internet].

[3] Dehlendorf C, Grumbach K, Schmittdiel JA, Steinauer J (2017). Shared decision making in contraceptive counseling. Contraception..

[4] Chen M, Lindley A, Kimport K, Dehlendorf C (2019). An in-depth analysis of the use of shared decision making in contraceptive counseling. Contraception..

[5] Washington State. Providing high quality, affordable health care to Washingtonians based on the recommendations of the blue ribbon commission on health care costs and access [Internet]. E2S SB 5930 2007. Available from: http://apps.leg.wa.gov/billinfo/summary.aspx?bill=5930&year=2007. Accessed 3 Aug 2015.

[6] Légaré F, Stacey D, Forest P-G, Coutu M-F (2011). Moving SDM forward in Canada: milestones, public involvement, and barriers that remain. Z Evid Fortbild Qual Gesundhwes.

[7] Coulter A, Edwards A, Elwyn G, Thomson R (2011). Implementing shared decision making in the UK. Z Evid Fortbild Qual Gesundhwes.

[8] Munro S, Kornelsen J, Wilcox E, Corbett K, Bansback N, Janssen P (2017). Do women have a choice? Care providers’ and decision makers’ perspectives on barriers to access of health services for birth after a previous caesarean. Birth..

[9] Munro S, Janssen P, Wilcox E, Corbett K, Bansback N, Kornelsen J (2017). Seeking control in the midst of uncertainty: women’s experiences of choosing mode of delivery after caesarean. Women Birth.

[10] Munro S, Kornelsen J, Wilcox E, Kaufman S, Bansback N, Corbett K, et al. Implementation of shared decision-making in healthcare policy and practice: a complex adaptive systems perspective. Evid Policy. 2019; 10.1332/174426419X15468571657773.

[11] Légaré F, Ratté S, Gravel K, Graham ID (2008). Barriers and facilitators to implementing shared decision-making in clinical practice: update of a systematic review of health professionals’ perceptions. Patient Educ Couns.

[12] Légaré F, O’Connor AM, Graham ID, Saucier D, Côté L, Blais J (2006). Primary health care professionals’ views on barriers and facilitators to the implementation of the Ottawa decision support framework in practice. Patient Educ Couns.

[13] Joseph-Williams N, Lloyd A, Edwards A, Stobbart L, Tomson D, Macphail S (2017). Implementing shared decision making in the NHS: lessons from the MAGIC programme. BMJ..

[14] Joseph-Williams N, Elwyn G, Edwards A (2014). Knowledge is not power for patients: a systematic review and thematic synthesis of patient-reported barriers and facilitators to shared decision making. Patient Educ Couns.

[15] Frosch DL, Singer KJ, Timmermans S (2011). Conducting implementation research in community-based primary care: a qualitative study on integrating patient decision support interventions for cancer screening into routine practice. Health Expect.

[16] Elwyn G, Scholl I, Tietbohl C, Mann M, Edwards AGK, Clay C (2013). “Many miles to go …”: a systematic review of the implementation of patient decision support interventions into routine clinical practice. BMC Med Inform Decis Mak.

[17] Scholl I, Hahlweg P, Lindig A, Bokemeyer C, Coym A, Hanken H (2018). Evaluation of a program for routine implementation of shared decision-making in cancer care: study protocol of a stepped wedge cluster randomized trial. Implement Sci.

[18] Tan ASL, Mazor KM, McDonald D, Lee SJ, McNeal D, Matlock DD (2018). Designing shared decision-making interventions for dissemination and sustainment: can implementation science help translate shared decision making into routine practice?. MDM Policy Pract.

[19] Thompson R, Manski R, Donnelly KZ, Stevens G, Agusti D, Banach M (2017). Right for me: protocol for a cluster randomised trial of two interventions for facilitating shared decision-making about contraceptive methods. BMJ Open.

[20] Cane J, O’Connor D, Michie S (2012). Validation of the theoretical domains framework for use in behaviour change and implementation research. Implement Sci.

[21] Atkins L, Francis J, Islam R, O’Connor D, Patey A, Ivers N (2017). A guide to using the theoretical domains framework of behaviour change to investigate implementation problems. Implement Sci.

[22] Michie S, van Stralen MM, West R (2011). The behaviour change wheel: a new method for characterising and designing behaviour change interventions. Implement Sci.

[23] Michie S, Johnston M, Abraham C, Lawton R, Parker D, Walker A (2005). Making psychological theory useful for implementing evidence based practice: a consensus approach. Qual Saf Health Care.

[24] Braun V, Clarke V (2006). Using thematic analysis in psychology. Qual Res Psychol.

[25] Elwyn Glyn, Rasmussen Julie, Kinsey Katharine, Firth Jill, Marrin Katy, Edwards Adrian, Wood Fiona (2016). On a learning curve for shared decision making: Interviews with clinicians using the knee osteoarthritis Option Grid. Journal of Evaluation in Clinical Practice.

[26] Scalia P, Elwyn G, Durand M-A (2017). “Provoking conversations”: case studies of organizations where Option Grid™ decision aids have become ‘normalized’. BMC Med Inform Decis Making.

[27] Fay M, Grande SW, Donnelly K, Elwyn G (2016). Using option grids: steps toward shared decision-making for neonatal circumcision. Patient Educ Couns.

[28] Donnelly KZ, Foster TC, Thompson R (2014). What matters most? The content and concordance of patients’ and providers’ information priorities for contraceptive decision making. Contraception..

[29] Wyatt KD, Branda ME, Anderson RT, Pencille LJ, Montori VM, Hess EP (2014). Peering into the black box: a meta-analysis of how clinicians use decision aids during clinical encounters. Implement Sci.

[30] Montori VM, Kunneman M, Brito JP (2017). Shared decision making and improving health care: the answer is not in. JAMA..

[31] Muscat DM, Shepherd HL, Morony S, Smith SK, Dhillon HM, Trevena L (2016). Can adults with low literacy understand shared decision making questions? A qualitative investigation. Patient Educ Couns.

[32] Muscat DM, Morony S, Smith SK, Shepherd HL, Dhillon HM, Hayen A (2017). Qualitative insights into the experience of teaching shared decision making within adult education health literacy programmes for lower-literacy learners. Health Expect.

[33] Shepherd HL, Barratt A, Jones A, Bateson D, Carey K, Trevena LJ (2016). Can consumers learn to ask three questions to improve shared decision making? A feasibility study of the ASK (AskShareKnow) patient-clinician communication model(®) intervention in a primary health-care setting. Health Expect.

[34] Sansoni JE, Grootemaat P, Duncan C (2015). Question prompt lists in health consultations: a review. Patient Educ Couns.

