# Supplementary material for: Investigation of factors influencing the implementation of two shared decision-making interventions in contraceptive care: a qualitative interview study among clinical and administrative staff
Source: Implement Sci. 2019 Nov 9;14:95. doi: 10.1186/s13012-019-0941-z (PMC6842477; doi:10.1186/s13012-019-0941-z)
Supplement: Supplementary file 1 — Additional file 1. Orientation materials for clinics implementing the Right For Me interventions. [file 13012_2019_941_MOESM1_ESM.pdf]

right for me

Decision Aids + Training

Introduction

This slide deck is intended to support people in your clinic (administrative staff, health professionals, and others) to implement the Right For Me Decision Aids + Training.

© 2016 Trustees of Dartmouth College

About

The Right For Me Decision Aids are a set of seven one-page decision aids on birth control methods. They are intended to be used by health professionals during the health care visit. The Decision Aids are available in English and Spanish.

The Right For Me Decision Aid Training consists of a four-minute Video and Frequently Asked Questions intended to be reviewed by health professionals before beginning to use the Decision Aids.

© 2016 Trustees of Dartmouth College

Objective

The aim of the Decision Aids is to support health professionals to facilitate shared decision-making about birth control methods with patients in the health care visit.

The aim of the Training Video and Frequently Asked Questions is to enhance health professionals' motivation, skills, and self-efficacy to use the Decision Aids.

© 2016 Trustees of Dartmouth College

Audience

The target audience for the Decision Aids + Training is any health professional who provides information or counseling about contraception to patients receiving health care in your clinic.

© 2016 Trustees of Dartmouth College

Development

The Decision Aids + Training were developed by researchers at Dartmouth College and their project partners, based on previous research and extensive consultation with patients, health professionals, and other stakeholders.

© 2016 Trustees of Dartmouth College

Implementation

Implementing the Decision Aids + Training is a team effort. The tasks involved are explained below and on the remaining slides.

What?	Who?
1. Supplying the materials	The Right For Me project team
2. Taking the Training	Health professionals
3. Using the Decision Aids	Health professionals
4. Other support tasks	Administrative staff, health professionals, and/or others

© 2016 Trustees of Dartmouth College

1. Supplying the materials

The Right For Me project team

© 2016 Trustees of Dartmouth College

Decision Aids

We have supplied your clinic with:

- Tear-pads of the seven different Decisions Aids in English and Spanish (each set is bound with a rubber band)
- 1 desktop or wall-mounted display stand for each exam room

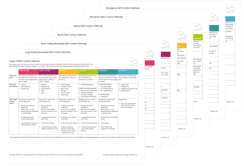

© 2016 Trustees of Dartmouth College

Training

We have supplied your clinic with online access to:

- The Training Video
- The Frequently Asked Questions

© 2016 Trustees of Dartmouth College

2. Taking the Training

For health professionals

© 2016 Trustees of Dartmouth College

Taking the Training

You can take the Training as often as you wish by:

1. Visiting www.rightforme.org
2. Navigating to 'For Clinics' and then 'Decision Aids + Training'
3. Entering the clinic username and password

Your Project Contact will give you the clinic username and password. Please do not share this information with others outside your clinic.

© 2016 Trustees of Dartmouth College

3. Using the Decision Aids

For health professionals

© 2016 Trustees of Dartmouth College

Using the Decision Aids

All guidance on using the Decision Aids is provided in the Training Video and Frequently Asked Questions.

© 2016 Trustees of Dartmouth College

4. Other support tasks

For administrative staff, health professionals, and/or others

© 2016 Trustees of Dartmouth College

Decision Aid Management

Please place tear-pads of the seven different Decision Aids on display in each exam room.

Please check periodically to make sure that each exam room has enough Decision Aids. If you are running low on Decision Aids in English and/or Spanish, please tell your Project Contact. They will ask us to send extra Decision Aids to your clinic.

© 2016 Trustees of Dartmouth College

Thank you. Good luck!

© 2016 Trustees of Dartmouth College

right for me

Video + Prompt Card

Introduction

This slide deck is intended to support people in your clinic (administrative staff, health professionals, and others) to implement the Right For Me Video + Prompt Card.

© 2016 Trustees of Dartmouth College

About

The Right For Me Video is a three-minute video intended to be viewed by patients immediately before the health care visit. It is viewed on an iPad with headphones. The Video is available in English and Spanish, with and without on-screen captions.

The Right For Me Prompt Card is a small card intended to be taken by patients immediately before the health care visit. The Prompt Card is available in English and Spanish.

© 2016 Trustees of Dartmouth College

Objective

The aim of the Video is to enhance patients' motivation, skills, and self-efficacy to ask health professionals three specific questions:

(1) What are my options?
(2) What are the pros and cons of those options?
(3) How likely are those pros and cons to happen to me?

The aim of the Prompt Card is to remind patients of the three questions presented in the video.

© 2016 Trustees of Dartmouth College

Audience

The target audience for the Video + Prompt Card is any patient waiting to see a health professional in your clinic (for example, while in the waiting room or after the rooming-in process).

© 2016 Trustees of Dartmouth College

Development

The Video + Prompt Card were developed by researchers at Dartmouth College and their project partners, based on previous research and extensive consultation with patients, health professionals, and other stakeholders.

© 2016 Trustees of Dartmouth College

Preview

You can preview the Video + Prompt Card by:

1. Visiting www.rightforme.org
2. Navigating to 'For Clinics' and then 'Video + Prompt Card'
3. Entering the clinic username and password

Your Project Contact will give you the clinic username and password. Please do not share this information with others outside your clinic.

© 2016 Trustees of Dartmouth College

Implementation

Implementing the Video + Prompt Card is a team effort. The tasks involved are explained below and on the remaining slides.

What?	Who?
1. Supplying the materials	The Right For Me project team
2. Facilitating patients viewing the Video	Administrative staff, health professionals, and/or others
3. Facilitating patients taking the Prompt Card	Administrative staff, health professionals, and/or others
4. Other support tasks	Administrative staff, health professionals, and/or others

© 2016 Trustees of Dartmouth College

1. Supplying the materials

The Right For Me project team

© 2016 Trustees of Dartmouth College

Right For Me Video

We have supplied your clinic with:

- 2 iPads programmed to view the Video
- 2 sets of headphones
- Cleaning wipes

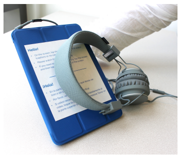

© 2016 Trustees of Dartmouth College

Right For Me Prompt Card

We have supplied your clinic with:

- 3 display stands of English Prompt Cards
- 1 display stand of Spanish Prompt Cards
- Extra refills of Prompt Cards

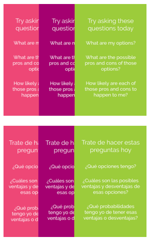

© 2016 Trustees of Dartmouth College

2. Facilitating patients viewing the Video

For administrative staff, health professionals, and/or others

© 2016 Trustees of Dartmouth College

Facilitating Patients Viewing the Video

We encourage you to facilitate patients viewing the Video in a way that works for your clinic. Things you may wish to think about include:

- Who will hand the iPads and headphones to patients?
- When and where will you hand the iPads and headphones to patients?
- What will you say to patients about the Video?
- Who will collect the iPads and headphones from patients?

The next slides provide some practice guidance that may be helpful.

© 2016 Trustees of Dartmouth College

Guidance for Clinic Staff

Before handing an iPad to a patient, open the cover. You should see four videos displayed on the screen.

If you see that a video is playing, ① tap anywhere on the screen and ② tap 'Done' in the top left corner. Close the cover.

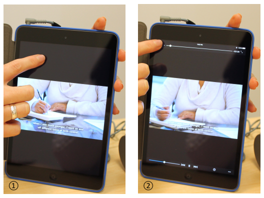

© 2016 Trustees of Dartmouth College

Guidance for Patients

Basic instructions for patients are provided on the iPad cover in English and Spanish.

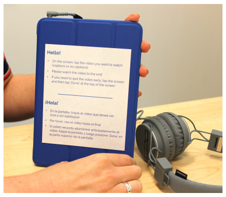

© 2016 Trustees of Dartmouth College

Guidance for Patients

Patients may choose to watch the Video in English or Spanish, with or without on-screen captions.

Patients should put on the headphones and ① tap the video they want to watch. It will play automatically.

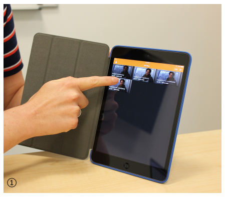

© 2016 Trustees of Dartmouth College

Guidance for Patients

If a patient wishes to exit a video before it has finished playing, they should ① tap anywhere on the screen and ② tap 'Done' in the top left corner.

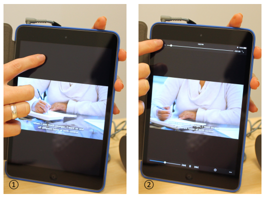

© 2016 Trustees of Dartmouth College

Guidance for Patients

If a patient wishes to increase or decrease the volume of a video, they should ① press the long up or down button on the right side of the iPad.

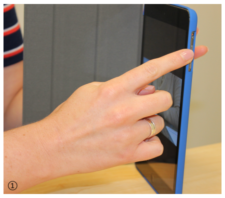

© 2016 Trustees of Dartmouth College

3. Facilitating patients taking the Prompt Card

For administrative staff, health professionals, and/or others

© 2016 Trustees of Dartmouth College

Facilitating Patients Taking the Prompt Card

We encourage you to facilitate patients taking the Prompt Card in a way that works for your clinic. Things you may wish to think about include:

- Will you hand the patient a Prompt Card when you give them the iPad?
- Will you put the Prompt Cards out on display and refer patients to them?
- What will you say to patients about the Prompt Card?

Patients may take the Prompt Card home with them to keep.

© 2016 Trustees of Dartmouth College

4. Other support tasks

For administrative staff, health professionals, and/or others

© 2016 Trustees of Dartmouth College

iPad Management

Please ensure the iPads and headphones are stored securely in the clinic when not in use.

Please charge the iPads as often as needed, including every night. Plug the iPad charger into a wall outlet (not a computer).

© 2016 Trustees of Dartmouth College

iPad Management

Please check periodically that the iPads remain locked into the Video application. To do this, press the iPad home button at the bottom of the iPad. When you press this, the screen should not change. If the screen changes, please tell your Project Contact.

© 2016 Trustees of Dartmouth College

Prompt Card Management

Please check periodically to make sure you have enough Prompt Cards. If you are running low on Prompt Cards in English and/or Spanish, please tell your Project Contact. They will ask us to send extra Prompt Cards to your clinic.

© 2016 Trustees of Dartmouth College

Thank you.
Good luck!

© 2016 Trustees of Dartmouth College
